# Imaging-guided cardiac resynchronization therapy lead placement in patients with congenitally corrected transposition of the great arteries

**DOI:** 10.1093/ehjimp/qyae083

**Published:** 2024-08-16

**Authors:** Luuk H G A Hopman, Frebus J van Slochteren, Thelma C Konings, Emanuele Rondanina, Cornelis P Allaart, Marco J W Götte, Vokko P van Halm

**Affiliations:** Department of Cardiology, Amsterdam UMC, De Boelelaan 1118, 1081 HV Amsterdam, The Netherlands; Department of Cardiology, UMC Utrecht, Utrecht, The Netherlands; Department of Cardiology, Amsterdam UMC, De Boelelaan 1118, 1081 HV Amsterdam, The Netherlands; Department of Bioengineering and Biomedical Engineering, Technische Universiteit Eindhoven, Eindhoven, The Netherlands; Department of Cardiology, Amsterdam UMC, De Boelelaan 1118, 1081 HV Amsterdam, The Netherlands; Department of Cardiology, Amsterdam UMC, De Boelelaan 1118, 1081 HV Amsterdam, The Netherlands; Department of Cardiology, Amsterdam UMC, De Boelelaan 1118, 1081 HV Amsterdam, The Netherlands

**Keywords:** cardiac resynchronization therapy, congenitally corrected transposition of the great arteries, imaging, heart failure, image integration, cardiac magnetic resonance imaging

**Figure:**
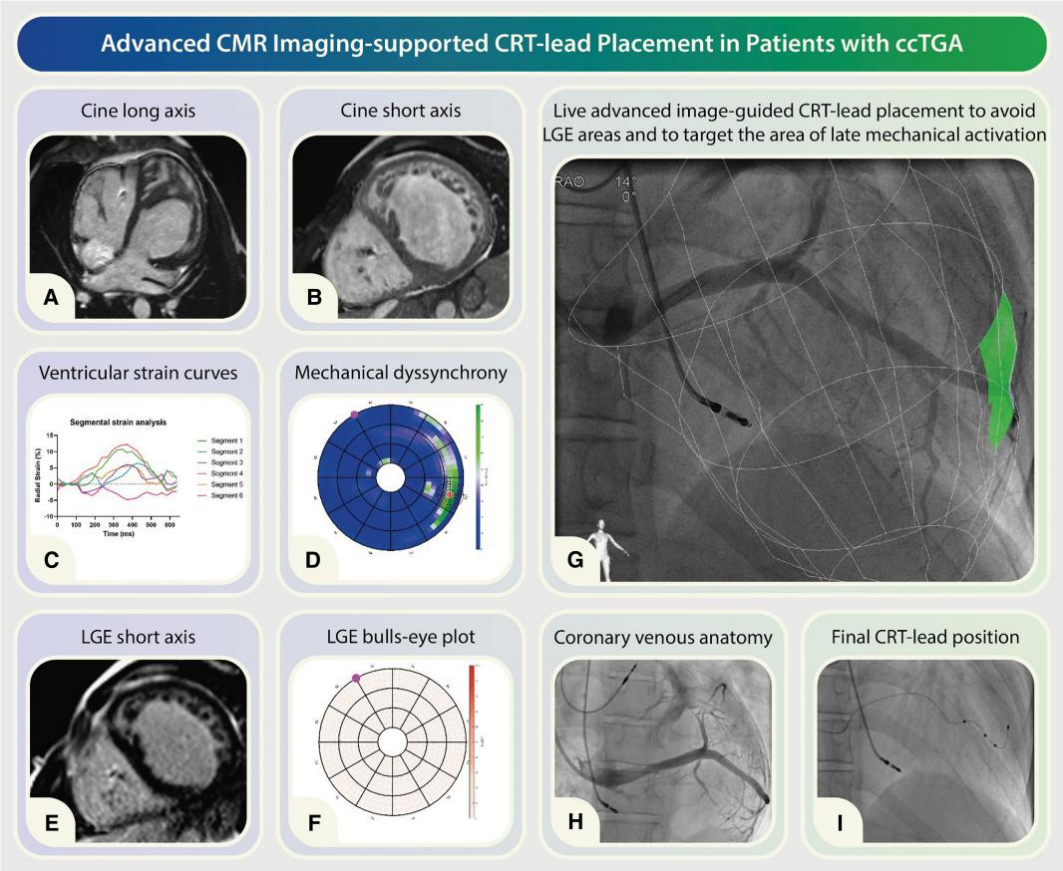


Congenitally corrected transposition of the great arteries (ccTGA) is a relatively uncommon congenital heart condition, comprising ∼0.5% of congenital heart defects. In ccTGA, the cardiac anatomy differs from the norm, with the right atrium (RA) connected to a morphological left ventricle (LV) on the right side, while the left atrium (LA) connects to a morphological right ventricle (RV) on the left side (*Panel A*). Typically, the morphological LV links to the pulmonary artery, and the morphological RV connects to the aorta. Despite being termed ‘corrected’, this configuration can lead to a variety of clinical symptoms and haemodynamic challenges due to accompanying anomalies commonly observed with ccTGA.

One of the inherent abnormalities associated with ccTGA is a cardiac conduction system disorder, which may manifest as progressive conduction block. As a result, a significant number of ccTGA patients require permanent pacemaker implantation to secure and regulate heart rhythm, and maintain adequate cardiac function. However, patients dependent on continuous ventricular pacing are at risk of developing pacing-induced ventricular dyssynchrony. This condition can lead to the further deterioration of systemic ventricular function and may progress to heart failure over time (*Panel B*). In some cases therefore, an upgrade to cardiac resynchronization therapy (CRT) may be necessary. Several studies have indicated that biventricular pacing can aid in preserving systemic ventricular function in ccTGA patients. A systematic review on CRT for the failing systemic RV by Kharbanda *et al.* indicated that CRT in this patient category can be an effective treatment, though supporting evidence is still limited. Moreover, a recent multicentre study found that patients with ccTGA who had previously undergone ventricular pacing showed improvements in QRS duration and New York Heart Association (NYHA) class 6 months after upgrading to CRT, but no increase in ejection fraction was observed. This lack of improvement in ejection fraction might be due to a lack of knowledge about optimal lead placement locations in ccTGA patients, which can result in diverse outcomes.

Traditionally, CRT involves the implantation of an additional ventricular lead to synchronize electrical activation across both ventricles, thereby restoring cardiac function. In ccTGA patients, the traditional LV lead will be placed over the morphological RV. This CRT lead can be placed surgically if the coronary sinus (CS) anatomy is inappropriate for transvenous CRT lead placement. Ideally, transvenous CRT lead placement is performed to avoid the need for general anaesthesia and sternotomy. In conventional CRT, targeting the lateral or posterolateral LV wall is standard for electrical resynchronization. However, in ccTGA patients, altered anatomy and haemodynamics pose significant challenges in determining the ideal site for CRT lead. To address this challenge in pre-procedural planning, echocardiography, cardiac magnetic resonance (CMR) imaging, and/or computed tomography (CT) can be performed for the assessment of cardiac function, scar tissue, and venous anatomy.

Given the highly variable and patient-specific nature of identifying the optimal pacing site in patients with ccTGA, adopting a personalized and targeted approach becomes crucial. Previous studies have emphasized the benefits of utilizing image-guided lead placement to improve clinical outcomes in the general CRT population. Echocardiography and CMR imaging emerge as pivotal tools in this endeavour, enabling precise localization of lead placement to the myocardial region exhibiting the latest mechanical activation. The underlying principle of CRT success lies in the electrical pre-excitation of the latest contracting segments, aiming to restore synchrony of ventricular contraction and thereby facilitating mechanical improvement. This underscores the significance of using contraction pattern imaging such as speckle tracking echocardiography or feature tracking strain on CMR to identify the optimal site for pacing. Utilizing CMR feature tracking analysis on high-temporal resolution short-axis cine images provides a robust method for quantifying myocardial deformation, offering advantages over speckle tracking echocardiography as this approach minimizes biases and operative variability, thereby facilitating the precise identification of the area of latest mechanical activation (*Panel C*). Feature tracking strain for this purpose involves calculating the segmental radial strain and monitoring its amplitude progression over time. This technique provides a detailed visual representation of the heart’s mechanical activation over time. The target area is then identified by locating where the latest and highest positive radial strain occurs. Due to inter-patient variability in the amplitude and timing of radial strain, there are no universal thresholds for these parameters. The visualization of these data in a 36-segment bull’s-eye plot format can provide a comprehensive insight into myocardial mechanics (*Panel D*).

It is critical to ensure that the region of latest mechanical activation does not consist of myocardial scar, as scarred tissue typically demonstrates impaired electrical propagation. Moreover, pacing within scar tissue is associated with CRT non-response, which may lead to an elevated risk of cardiovascular death or hospitalization for heart failure; hence, avoiding such placement is paramount. Late gadolinium enhancement (LGE) CMR imaging is the gold standard for identifying myocardial scar. The integration of LGE information into a separate bull’s-eye plot allows for visualization of scar distribution (*Panel E*). The synergy between bull’s-eye plots depicting mechanical activation and scar distribution serves as a valuable tool in identifying the optimal ventricular pacing site (target area) (*Panel F*).

During the implantation procedure, real-time visualization of the target area can be achieved through the co-registration of the predefined target area onto the fluoroscopy images as facilitated by the CARTBox-Suite platform (CART-Tech B.V., Utrecht, The Netherlands) (*Panel G*). The visualization of the target for lead deployment may enhance the likelihood of optimal pacing, as operators endeavour to position the ventricular lead as close as possible to the target area in a patient-specific manner (*Panels H* and *I*).

For CRT lead implantation procedures, understanding the venous anatomy is essential to anticipate ventricular lead placement. This is particularly mandatory in cases of ccTGA, where the cardiac anatomy presents unique challenges. Typically, the CS aligns with the morphologic atria, often draining into the RA, while venous branches, such as the great cardiac vein and lateral branches, develop alongside the morphologic ventricles. Consequently, the venous structures draining the morphologic RV tend to be small and restricted in length, often failing to extend fully from the RV apex, which may complicate lead implantation procedures. Due to the complexity of this vascular anatomy, the use of pre-procedural imaging emerges as an important strategy for effective lead placement.

CT imaging conducted prior to implantation can be used for assessing CS access and delineating the course of venous branches. By providing detailed anatomical information, CT scans facilitate the anticipation of potential obstacles and the formulation of appropriate navigation strategies during the procedure. Additionally, contrast-enhanced magnetic resonance angiography (MRA) obtained through CMR imaging is another viable option for preoperative venous anatomy evaluation. While CMR can offer excellent visualization of CS access, its lower spatial resolution may present challenges in accurately tracing the branches. Utilizing 3D models derived from CT or CMR scans of venous branches prior to CRT lead implantation in patients with ccTGA and real-time visualization thereof in conjunction with the (live) fluoroscopy can significantly support the implantation procedure, thereby improving the precision and efficacy of lead placement. However, it remains unknown whether different lead placement strategies yield different outcomes specifically in ccTGA patients. Additionally, potential complications directly associated with the imaging techniques require further investigation. While CT and CMR provide excellent anatomical details, real-time lead placement relies on fluoroscopy, which can result in discrepancies between pre-procedure imaging and intra-procedure fluoroscopic findings. As imaging-guided CRT lead placement in ccTGA patients is still being explored, studies focusing on long-term outcomes, limitations, and potential complications are necessary.

In conclusion, achieving optimal CRT lead placement in ccTGA patients presents a significant challenge owing to their altered cardiac anatomy, underscoring the necessity of personalized strategies. CMR imaging emerges as a crucial tool in this effort, enabling precise identification of the ideal pacing site through the analysis of latest mechanical activation area and ventricular scar identification. Incorporating CMR or CT into procedural planning and real-time visualization thereof in conjunction with (live) fluoroscopy facilitates the evaluation of venous anatomy, enhancing the precision of lead placement and ultimately leading to improved clinical outcomes for ccTGA patients undergoing CRT. Further investigation is warranted to comprehensively clarify the enduring impacts of imaging-guided CRT lead placement in patients with ccTGA.

## Notes

**Consent:** Written informed consent was obtained from the patient for publication of this case report in line with COPE guidance.

**Funding:** None declared.

**Data availability:** No new data were generated or analysed in support of this research.

## Lead author biography

Luuk Hopman is a postdoctoral researcher at the Department of Cardiology at Amsterdam UMC, where he specializes in cardiovascular magnetic resonance imaging (CMR) and electrophysiology. Over the past 5 years, his PhD research has centred on the in-depth study of CMR imaging in patients with atrial fibrillation, focusing particularly on atrial myopathy. Beyond this, Luuk has explored cardiac responses to various pacing strategies using advanced CMR imaging techniques. In the past 2 years, he has made significant contributions to the field of interventional MRI. His work includes performing real-time MRI-guided right heart catheterizations and flutter ablation procedures directly within the MRI scanner.

